# Navigating fluoroquinolone resistance in Gram-negative bacteria: a comprehensive evaluation

**DOI:** 10.1093/jacamr/dlae127

**Published:** 2024-08-14

**Authors:** Linda Kherroubi, Joanna Bacon, Khondaker Miraz Rahman

**Affiliations:** School of Cancer and Pharmaceutical Science, King’s College London, London SE1 9NH, UK; Discovery Group, Vaccine Development and Evaluation Centre, UK Health Security Agency, Porton Down, Salisbury SP4 0JG, UK; School of Cancer and Pharmaceutical Science, King’s College London, London SE1 9NH, UK

## Abstract

Since the introduction of quinolone and fluoroquinolone antibiotics to treat bacterial infections in the 1960s, there has been a pronounced increase in the number of bacterial species that have developed resistance to fluoroquinolone treatment. In 2017, the World Health Organization established a priority list of the most critical Gram-negative resistant pathogens. These included *Klebsiella pneumoniae*, *Acinetobacter baumannii*, *Pseudomonas aeruginosa*, and *Escherichia coli*. In the last three decades, investigations into the mechanisms of fluoroquinolone resistance have revealed that mutations in the target enzymes of fluoroquinolones, DNA gyrase or topoisomerase IV, are the most prevalent mechanism conferring high levels of resistance. Alterations to porins and efflux pumps that facilitate fluoroquinolone permeation and extrusion across the bacterial cell membrane also contribute to the development of resistance. However, there is a growing observation of novel mutants with newer generations of fluoroquinolones, highlighting the need for novel treatments. Currently, steady progress has been made in the development of novel antimicrobial agents that target DNA gyrase or topoisomerase IV through different avenues than current fluoroquinolones to prevent target-mediated resistance. Therefore, an updated review of the current understanding of fluoroquinolone resistance within the literature is imperative to aid in future investigations.

## Antimicrobial resistance in Gram-negative bacteria

The growing use of antibiotics in the last century, since their discovery, has led to the emergence of antibiotic-resistant bacteria, which pose a great danger to public health. As current antibiotic treatments become rapidly ineffective in treating infections due multi-drug resistant (MDR) bacteria, the demand to develop novel antibiotics has surged. Unfortunately, there has been slow progress in the successful development of novel antibiotics over the last few decades. Reduced funding, initiative, and interest from pharmaceutical companies have resulted in a dry clinical pipeline for the development of new antibiotics to combat antimicrobial resistance (AMR).^1^

AMR develops within a small population of bacteria that evade antibiotic action. This trait can be either natural or acquired. Natural resistance is a protective genetic mechanism induced by the presence of antibiotics or is inherently active to confer resistance upon antibiotic exposure. Acquired resistance, on the other hand, involves the acquisition of resistant genes through horizontal gene transfer or chromosomal mutations, which confer antibiotic resistance.^2^ There are four main mechanisms of AMR, these include preventing antibiotic uptake (i), antibiotic inactivation (ii), antibiotic efflux (iii), all of which are naturally intrinsic modes of resistance. The fourth mechanism refers to the modification of the antibiotic's cellular target (iv), which is an acquired resistance.^2^

The ESKAPE class (*Enterococcus spp*., *Staphylococcus aureus*, *Klebsiella pneumonia*, *Acinetobacter baumannii, Pseudomonas aeruginosa*, *Enterobacter* species) are of serious concern due to the major threat they pose to the spread of AMR.^3,4^ Among these, Gram-negative bacteria exhibit higher rates of AMR compared with Gram-positive bacteria. This is primarily due to the additional resistance conferred by the outer membrane, a feature unique to Gram-negative bacteria.^3,5,6^  *Pseudomonas aeruginosa* is a major contributor to nosocomial infections, particularly at surgical sites or in the urinary tract, and it has exhibited a 15%–25% increase in resistance to antibiotic classes such as carbapenems and fluoroquinolones. *Acinetobacter baumannii*, responsible for nosocomial urinary-tract infections (UTIs) and respiratory infections, has shown increasing antibiotic resistance with a 2007 report from an American ICU showing only 10% of isolates were susceptible to cephalosporins or fluoroquinolones.^7,8^  *Klebsiella pneumoniae* targets the urinary, respiratory, and bloodstream systems, and like *A. baumannii*, it is an opportunistic pathogen that targets immunocompromised individuals. *Klebsiella pneumoniae* has exhibited resistance to multiple antibiotic classes, including penicillins, cephalosporins, and fluoroquinolones, with global cephalosporin resistance exceeding 30%.^1^ Pathogenic forms of *Escherichia coli* can cause food poisoning and UTIs. This species has exhibited resistance across most antibiotic classes, including fluoroquinolones.^1,8^

### Fluoroquinolones

**Figure 1.**
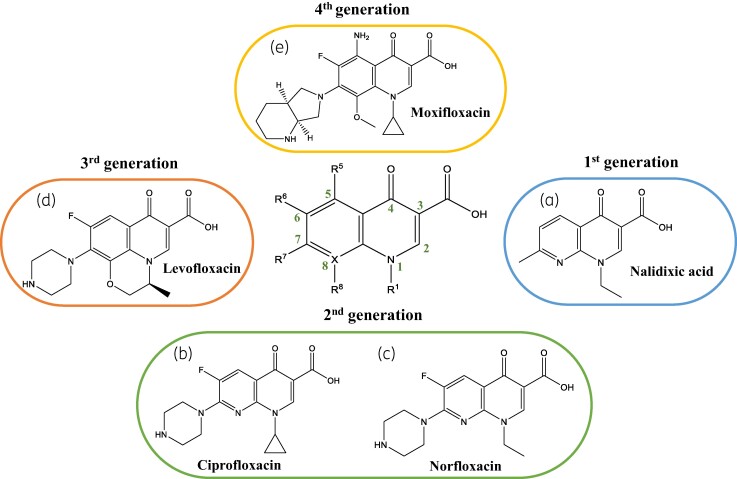
Skeletal structure of the core fluoroquinolone includes a bicyclic ring with carbonyls present on the C3 and C4 positions. Substitutions on the core structure generate different generations of fluoroquinolones: first generation nalidixic acid (a), second generation ciprofloxacin (b) and norfloxacin (c), third generation levofloxacin (d), and fourth generation moxifloxacin (e). Figure created with ChemDraw.

Fluoroquinolones originated as synthetic antimicrobials, and since the introduction of the first quinolone, nalidixic acid, in the early 1960s, this class has undergone modifications over four generations to improve the efficacy of antibacterial activity and broaden the spectrum of bacteria they target.^9^ Nalidixic acid was first identified with moderate antibacterial activity against most Gram-negative bacteria, except *P. aeruginosa.* Subsequent modifications led to second generation fluroquinolones, such as ciprofloxacin and norfloxacin, which were active against *P. aeruginosa* due to a fluorine substitution to C6 of the quinolone scaffold which strengthened pi-stacking interactions formed between the fluoroquinolone and the DNA bases.^10^ These changes increased efficacy, reduced toxicity, and lowered susceptibility to single point mutations which conferred resistance in the first generation.^9,11^ Further modifications produced third generation fluoroquinolones, such as levofloxacin, and fourth generation, such as moxifloxacin, which offered continued improvements in efficacy and reduced toxicity (Figure 1).^9,11^ There are several fluoroquinolones that are actively prescribed worldwide. In the UK, the four available fluoroquinolones are ciprofloxacin, levofloxacin, moxifloxacin, and ofloxacin.^12^ Table 1 depicts some of the currently marketed fluoroquinolones and those in Phase III clinical trials.^13–29^ According to the 2021 report from the UK Health Security Agency, fluoroquinolones account for 2.9% of prescribed antibiotics in the UK, with ciprofloxacin representing 73.2% of the prescriptions.^30^

**Table 1.** Clinical use, antibacterial activity, pharmacokinetics/pharmacodynamics, and mode of administration of currently marketed fluoroquinolones and those in Phase III clinical trials

Fluoroquinolone	Clinical use	Activity against ESKAPE pathogens	Pharmacokinetics/ Pharmacodynamics	Mode of administration
**Currently marketed fluoroquinolones**				
Ciprofloxacin	Bone and joint infections, respiratory tract infections, UTIs, superficial bacterial eye infections, among others	*E. faecium* *S. aureus* *K. pneumoniae* *P. aeruginosa* *E. coli*	C_max_ = 3.5 mg/L Area under curve = 30 mg.h/L Half-life = 4h Protein binding = 20%–40% Volume of distribution = 2–3.04L/kg Oral bioavailability = 70%–80% Total clearance = 9.62 mL/min*kg	IV, PO
Delafloxacin	Acute bacterial skin and skin structure infections	*S. aureus* *K. pneumoniae* *P. aeruginosa* *E. coli*	C_max_ = 7.5 mg/L Area under curve = 23.4 mg.h/L Half-life = 4.2–8.5 h Protein binding = 84% Volume of distribution = 30–48L Oral bioavailability = 58.8% Total clearance = 16.3L/h	IV, PO
Levofloxacin	Eye infections, respiratory tract infections, UTIs, skin, and soft-tissue infections, among others	*S. aureus* *K. pneumoniae* *P. aeruginosa* *E. coli* If *P. aeruginosa* is the confirmed or suspected pathogen, it is advised to use combination therapy with an anti-pseudomonal β-lactam	C_max_ = 2.8 mg/L Area under curve = 4.78 mg.h/L Half-life = 6–8 h Protein binding = 24%–38% Volume of distribution = 89–112 L Oral bioavailability = 99% Total clearance = 8.64–13.56 L/h	IV, PO, inhalation
Moxifloxacin	Eye infections, sinusitis, respiratory tract infections, skin, and soft-tissue infections, among others	*S. aureus* *K. pneumoniae* *E. coli*	C_max_ = 3.1 mg/L Area under curve = 30 mg.h/L Half-life = 13h Protein binding = 50% Volume of distribution = 1.7–2.7 L/kg Oral bioavailability = 90% Total clearance = 12L/hr	IV, PO
Ofloxacin	Eye infections, UTIs, skin, and soft-tissue infections, among others	*S. aureus* *K. pneumoniae* *P. aeruginosa* *E. coli*	C_max_ = 4.8 mg/L Area under curve = 64 mg.h/L Half-life = 6h Protein binding = 40% Volume of distribution = NA Oral bioavailability = 98% Total clearance = NA	IV, PO
**Fluoroquinolones currently in Phase III trials**				
EMROK/ EMROK O^a^	Skin infection, respiratory tract infections, pyelonephritis, among others	*S. aureus* *K. pneumoniae* *P. aeruginosa* *A. baumannii* *E. coli*	C_max_ = 4 mg/L Area under curve = 54 mg.h/L Half-life = NA Protein binding = NA Volume of distribution = NA Oral bioavailability = 90% Total clearance = NA	IV, PO

C_max_, maximum serum concentration a drug can achieve; h, hour; IV, intravenous; kg, kilogram; L, litre; mg, milligram; NA, not applicable; PO, oral.

The primary target enzyme of each fluoroquinolone listed in this table is bacterial type II topoisomerase.

^a^Also known as levonadifloxacin/alalevonadifloxacin).^13–29^

### Mechanism of action of fluoroquinolones

DNA gyrase is the primary target of fluoroquinolones, while topoisomerase IV serves as a secondary target.^2,31^ However, the primary target enzyme can vary between fluoroquinolones and bacterial species which complicates the synthesis of fluoroquinolones and limits their spectrum of activity against bacteria.^32,33^

**Figure 2.**
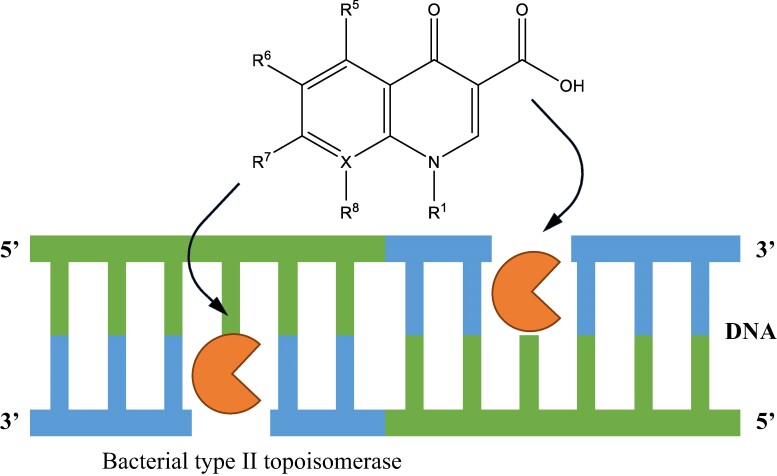
Two fluoroquinolone molecules intercalate between the unpaired bases of DNA cleaved by DNA gyrase/topoisomerase IV to stabilize the enzyme-DNA cleavage. This ternary complex (enzyme-DNA-fluoroquinolone) inhibits DNA gyrase/topoisomerase IV activity such that it can no longer induce strand relaxation or negative supercoils.

**Figure 3.**
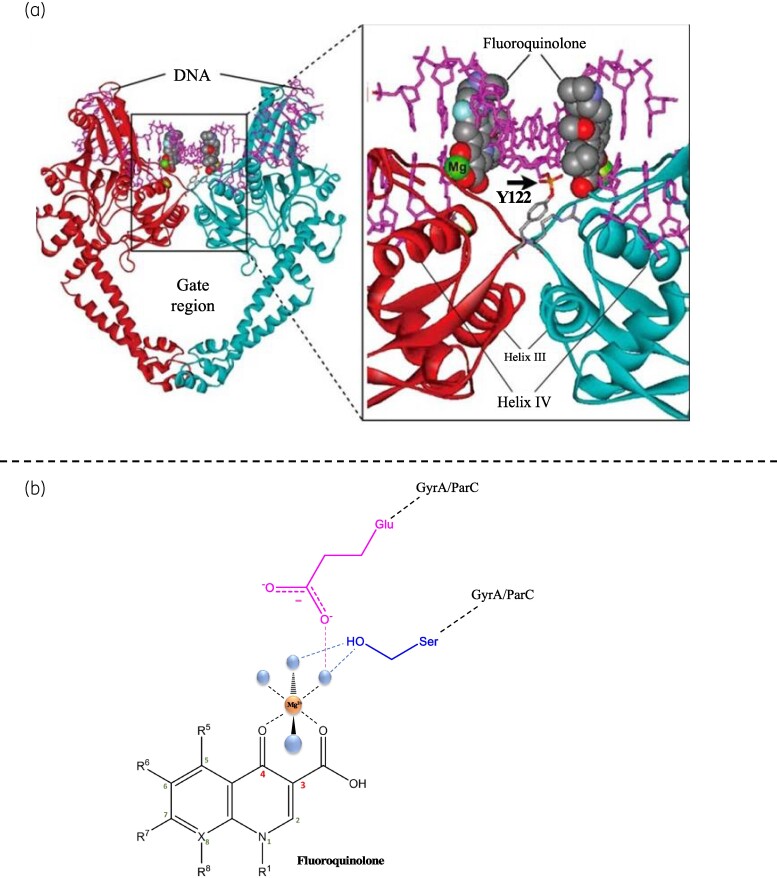
(a) 3D crystal structure of *Acinetobacter baumannii* topoisomerase IV complexed with DNA (purple) and 2 fluoroquinolone molecules, moxifloxacin (grey space filling representation). The DNA gate region is zoomed in on the right-hand side, the black arrow shows the interaction between the tyrosine residue and DNA.^36^ (b) C3 and C4 carbonyls on the fluoroquinolone form a bridging interaction to a Mg^2+^ ion forming an octahedral complex with four additional water molecules, two water molecules interact with serine and aspartic acid residues in GyrA or in ParC. Figure created with ChemDraw.

Sequential and structural differences between bacterial and eukaryotic topoisomerases ensure that only the bacterial DNA gyrase and topoisomerase IV, in Gram-negative bacteria, are selectively targeted in the design of fluoroquinolones.^10,32,34^ Fluoroquinolones bind and stabilize the complex formed between the cleaved DNA and the type II topoisomerase enzyme. This binding prevents the disassociation of the enzyme-DNA cleavage complex and blocks the ligation process, resulting in the accumulation of these complexes in the cytoplasm.^10,32^ Specifically, fluoroquinolones bind non-covalently to the interface between the gate segment and the DNA binding region of the enzyme (Figure 2).^35^ X-ray crystallography studies of type II topoisomerases-DNA-fluoroquinolone complexes have revealed that two fluoroquinolone molecules bind, via Van der Waals and π-π stacking interactions, at the interface between the gate segment and the DNA binding domain of the target enzyme. The intertwinement of the two fluoroquinolone molecules between the unpaired bases on the scissile bonds forms a ternary complex involving DNA, the target enzyme, and the fluoroquinolone (Figure 3a).^10,36^ The C3 and C4 carbonyl groups present on the fluoroquinolone play a crucial role in mediating a bridging interaction between water and a divalent metal ion, typically a magnesium ion. Specifically, the C3 and C4 carbonyl groups interact with Mg^2+^ to form an octahedral complex involving four water molecules. Additionally, two water molecules interact with serine and aspartic acid residues in GyrA or homologous residues in ParC (Figure 3b).^10,36^ This interaction stabilizes the ternary complex and appears to be the primary connection between fluoroquinolones and bacterial type II topoisomerases.^37,38^ The interactions that form the ternary complex render the target enzyme ineffective, causing it to stall on the DNA and lose its ability to induce strand relaxation or negative supercoiling. As a result, the bacterial cell undergoes cell death, but the manner in which this occurs depends on how the ternary complex is processed. If the complex is not processed, cellular machinery involved in DNA transcription and replication becomes blocked, leading to inhibited bacterial cell growth and a slow death.^10^ On the other hand, if the target enzyme dissociates from the complex, the broken DNA strands cannot be ligated, resulting in chromosomal fragmentation and rapid cell death. This is supported by observations of chromosomal fragmentation in bacterial cells exposed to fluoroquinolones.^39^

The broad-spectrum efficacy of fluoroquinolones makes them an important class of antibiotics as they are a useful treatment option against a wide range of bacterial infections. Their importance could extend further as a carbapenem-sparing strategy, however, there is limited evidence that suggests fluroquinolones can serve as carbapenem-sparing agents in combating carbapenem-mediated resistance.^40^ A Taiwanese study demonstrated the efficacy of levofloxacin and ciprofloxacin as a carbapenem-sparing strategy against ESBL-producing *E. coli* or *K. pneumoniae*. Amongst 299 patients, both ciprofloxacin and levofloxacin demonstrated a lower 3-day mortality rate (8.3%) compared with patients treated with carbapenem therapy (23.3%).^40–42^ Although these findings support the possible use of fluoroquinolones as an oral alternative to carbapenems in the treatment of ESBL-bacteraemia, PMQR is commonly associated with ESBL genes which hinders the effectiveness of fluoroquinolones in treating ESBL-bacteraemia. As well as a carbapenem alternative, fluoroquinolones are a commonly frequented choice of oral switch therapy due to their high bioavailability.^43^ Oral switch therapy as part of a wider stewardship programme has been shown to reduce the length of hospital stay and financial expenditure.^44^

Although fluoroquinolones are an important class of antibiotics used for a wide variety of infections, the Food and Drug Administration (FDA) has placed prescribing restrictions due to adverse events. These include tendinopathy, peripheral neuropathy, aortic aneurysms, and tendon rupture.^45,46^ Although these treatment-related side effects are rare, their systematic use can have significant and debilitating effects on a patient’s quality of life. As such, the FDA has advised prescribers to offer alternative antibiotics for patients that present with uncomplicated UTIs, acute sinitis, or bronchitis and instead reserve fluroquinolone for patients who do not have alternative treatment options.^45,47^

## Mechanism of AMR to fluoroquinolones

**Figure 4.**
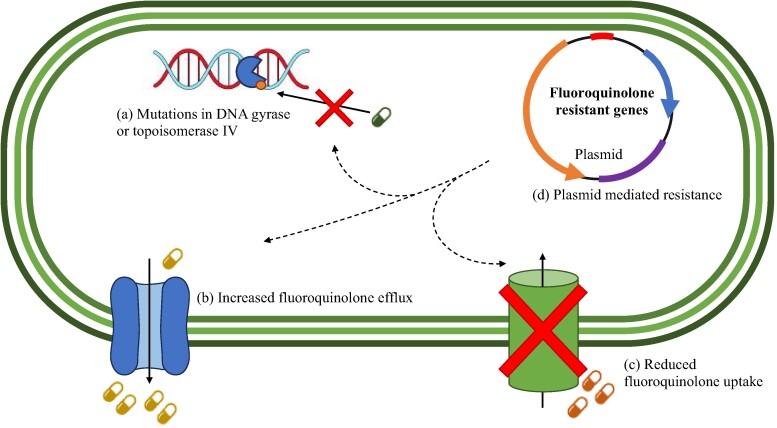
Mechanism of fluoroquinolone resistance in Gram-negative bacteria can be classified into three key groups. Mutations in the target enzymes, DNA gyrase or topoisomerase IV (a), alteration to efflux pumps (b) and porins (c) which control the transport of fluoroquinolones or PMQR (d).

Treatment with fluoroquinolones is becoming increasingly difficult, as reports indicate that this class is unable to effectively clear UTIs in 50% of cases.^48^ Commonly used fluoroquinolones like levofloxacin and ciprofloxacin have shown lower response rates against Gram-negative bacteria, including *E. coli*, *K. pneumoniae*, *A. baumannii*, and *P. aeruginosa*, compared with other antibiotics used in American ICUs.^7^ The increasing number of resistant cases against these pathogens prompted the WHO to prioritize the preservation of fluoroquinolones, considering their effectiveness in treating infections.^9^ The main mechanisms of fluoroquinolone resistance in Gram-negative bacteria involve mutations in the target enzymes, DNA gyrase and topoisomerase IV, as well as modifications in efflux pumps, porins, and plasmid-mediated quinolone resistance (PMQR) (Figure 4).^10^

### Mutations in DNA gyrase and topoisomerase IV

Mutations in the target enzymes, DNA gyrase and topoisomerase IV, are the predominant mechanism of resistance to fluoroquinolones.^49,50^ Single amino acid substitutions commonly occur between residues 67 and 106 in GyrA (using *E. coli* numbering), which constitute the quinolone resistance-determining region (QRDR). Among the numerous mutations reported in GyrA, the most prevalent mutations within the QRDR are observed at serine 83 (S83) and aspartic acid 87 (D87).^49^ A conserved motif consisting of four amino acids separating S83 and D87 forms a canonical dyad, and mutations within this dyad have been reported in various Gram-negative resistant species.^51,52^ The key mutation identified in the QRDR of ParC at the 80th position has been found to contribute to fluoroquinolone resistance in Gram-negative bacteria. Depending on the species, this mutation is primarily reported as a substitution from serine to either leucine (S80L) or isoleucine (S80I).^53^ It is important to note that amino acid numbering can vary between species and different papers may adopt different numbering systems, although the mentioned mutations are homologous. For the sake of clarity, this review will use *E. coli* numbering as the standard.

### Plasmid-mediated quinolone resistance

Bacterial plasmids are self-duplicating, independent genetic entities that can contain between 3 and 300 genes. Initially, plasmids that were found to confer antibiotic resistance were referred to as ‘R factors’, but since their discovery in the 1950s, they are more commonly known as PMQR plasmids, as they can confer resistance to multiple antibiotic classes.^54^

The discovery of the pMG252 plasmid in the *K. pneumoniae* UAB1 strain, which confers ciprofloxacin resistance, provided the first confirmation that plasmids can mediate quinolone resistance. This phenomenon has since been observed in other *K. pneumoniae* strains as well as in *E. coli*, with pMG252 enhancing resistance to various quinolones such as norfloxacin, levofloxacin, nalidixic acid, trovafloxacin, clinafloxacin, and pefloxacin by 4- to 16-fold.^54,55^ Further investigation involving the cloning and sequencing of pMG22 revealed the presence of the qnr gene, which is responsible for PMQR. There are multiple qnr genes located on plasmids, including qnrA, qnrS, and qnrB, which encode for Qnr proteins.^10^

Another mechanism of PMQR is related to the *AAC (6’)-Ib-cr* gene, which encodes a mutated protein called 6′-*N*-acetyltransferase (AAC (6’)-Ib-cr). This mutated protein, resulting from single amino acid substitutions W102R and D179YT, acquires acylating ability that it did not possess previously. AAC (6’)-Ib-cr can acylate fluoroquinolones, leading to their deactivation. The W102R mutation allows the C3 and C4 atoms of fluoroquinolones to interact with AAC (6’)-Ib-cr through hydrogen bonding, while the D179Y mutation enhances this interaction by forming pi-stacking interactions within the complex. These mutations facilitate the formation of the complex and negatively impact the binding of fluoroquinolones to their target enzymes.^56^ Consequently, the complex formation alters the binding of fluoroquinolones to their target enzymes, contributing to resistance.^10^

ESBLs and plasmid-mediated AmpC β-lactamase (pAmpC) genes can be propagated through mobile genetic elements, which also harbour PMQR genes. β-lactamase-producing isolates, such as *E. coli and K. pneumoniae,* that acquire PMQR genes can subsequently develop fluoroquinolone resistance.^57^ Over recent decades, the co-existence of PMQR and ESBL genes in *Enterobacteriaceae* has increased, primarily due to the horizontal transfer of resistant plasmids.^58^ Reports have shown a high prevalence of PMQR genes, such as *qnrB19*, in conjunction with β-lactamase genes such as bla_CTX-M_ in ESBL/AmpC-producing *Enterobacterales*.^59^ Additionally, reports increasingly describe the prevalence of plasmid-mediated resistance mechanisms for AmpC β-lactamase bla_CMY-2_, along with carbapenemases such as bla_NDM-1_, bla_OXA-48_, and bla_KPC-2_.^60^ Moreover, the widespread horizontal gene transfer among bacterial populations facilitates the spread of these resistance genes across different environments and hosts, including between animals and humans, raising significant public health concerns.^57^

### Alterations in efflux pumps

Efflux pumps, transporter proteins embedded within the bacterial cell membrane, help expel antibiotics from the cytosol, with different pumps exhibiting specificity towards different classes of antibiotics. Changes in the expression of efflux pumps can lead to increased extrusion of fluoroquinolones, reducing their accumulation within the cytosol.^49^ Among the five major families of efflux pumps found in Gram-negative bacteria, the Resistance-Nodulation-Division (RND) family is most commonly associated with fluoroquinolone resistance in Gram-negative bacteria.^61–65^ Upregulation of RND structural or global regulatory genes contributes to the development of fluoroquinolone resistance.

### Alterations in drug permeation

Porins are protein channels in bacterial cell membranes that facilitate the transport of molecules into the cytosol. While less studied than other resistance mechanisms in Gram-negative bacteria, they are thought to contribute to fluoroquinolone resistance through alterations in structural or regulatory proteins. The most extensively studied porins include OmpF in *E. coli*, OprF in *P. aeruginosa*, OmpA in *A. baumannii*, and OmpK35/OmpK36 in *K. pneumoniae.*^66^

### Escherichia coli

#### Mutations in DNA gyrase and topoisomerase IV

The single amino acid substitution from serine 83 to leucine 83 (S83L) was first reported in the *E. coli* strain 227 by Cullen et al. (1989), which exhibited high-level resistance to nalidixic acid. This mutation was later recognized as the most prevalent mutation in QRDR of GyrA, contributing to fluoroquinolone resistance.^52,67^ In a study analysing 74 clinically resistant *E. coli* isolates to ciprofloxacin, moxifloxacin, ofloxacin, and levofloxacin, it was found that 95% of the resistant isolates harboured the S83L mutation, while this mutation was not observed in five fluoroquinolone-susceptible isolates.^68,69^ S83L has also been identified in ciprofloxacin-resistant *E. coli* isolates obtained from ecological and poultry settings. This mutation conferred resistance to ciprofloxacin, as evidenced by a 16-fold increase in the MIC of ciprofloxacin compared with the control strain ATCC 25922.^70–72^ Additionally, the D87N mutation present in the QRDR of GyrA has been associated with high levels of fluoroquinolone resistance in ciprofloxacin-resistant *E. coli* isolates.^71,73^ The canonical dyad formed between S83 and D87 often occurs as a double mutation, leading to increased MIC values of fluoroquinolones compared with single mutations in the QRDR.^50,68^ These findings collectively underscore the role of S83L and D87N mutations acting together to confer fluoroquinolone resistance in *E. coli*. Moreover, the identification of resistance in *E. coli* isolates from various settings highlights the escalating spread of fluoroquinolone resistance in this species.^70^

Using DNA sequencing, researchers have identified the most common mutation in *parC* gene, which is the S80I mutation. This mutation was found in 95% of the 74 *E. coli* isolates that exhibited resistance to ciprofloxacin, moxifloxacin, levofloxacin, and ofloxacin.^68^ More recently, this mutation has also been identified in *E. coli* originating from swine waste. This finding is concerning as fluoroquinolones are frequently used to treat bacterial infections in livestock, but their decreasing effectiveness increases the likelihood of spread into humans.^74^

#### Plasmid-mediated quinolone resistance

Investigations have been conducted to identify the presence of *qnrA*, *qnrB*, and *qnrS* genes involved in PMQR in 200 clinical isolates of *E. coli*. Preliminary antimicrobial susceptibility assays were performed to identify isolates that were non-susceptible to fluoroquinolones.^75^ Results showed that 68% of the *E. coli* isolates demonstrated resistance to nalidixic acid, gatifloxacin, ciprofloxacin, levofloxacin, or norfloxacin. However, *qnrA* and *qnrB* genes were not detected, and only four isolates contained the *qnrS* gene. Researchers concluded that the low levels of these genes were likely since qnr genes commonly cause low levels of resistance, whereas the isolates in this study exhibited intermediate to high levels of resistance. They suggested that other mechanisms of resistance were likely involved in conferring fluoroquinolone resistance in these isolates. Unfortunately, this study did not assess other mechanisms of resistance.^75^ In a separate study conducted in 2017, researchers introduced *qnrS* and *qnrB* genes onto the chromosome of *E. coli* to investigate their ability to confer resistance. The presence of *qnrS* resulted in ciprofloxacin resistance, with the MIC surpassing the EUCAST breakpoint for ciprofloxacin resistance. This study highlighted *qnrS* as a determinant of resistance due to its ability to increase the MIC of ciprofloxacin without affecting the fitness of the bacterium.^76^

The existence of the *AAC (6’)-Ib-cr* gene has been detected over the past two decades. An observational study conducted between 1991 and 1997 found no expression of *AAC (6’)-Ib-cr* in intermediate or fully ciprofloxacin-resistant *E. coli* isolates.^77^ However, between 1998 and 2005, 7.1% of ciprofloxacin-resistant isolates were found to express *AAC (6’)-Ib-cr.* This indicates an increase in the prevalence of *AAC (6’)-Ib-cr* over the course of a decade, highlighting its growing importance as a contributor to ciprofloxacin resistance in *E. coli*. *AAC (6’)-Ib-cr* can enhance the MIC of ciprofloxacin by up to 16-fold.^77,78^

To demonstrate the impact of the *AAC (6’)-Ib-cr* gene, researchers introduced a vector containing *AAC (6’)-Ib-cr* into the *E. coli* strain ATCC 25922, which already contained chromosomal-mediated mutations such as S83L and D87N in DNA gyrase.^72,77,78^ The introduction of *AAC (6’)-Ib-cr* resulted in a 4- to 8-fold increase in the MIC of norfloxacin and ciprofloxacin. Furthermore, bactericidal activity was assessed using kill time curves, which revealed that strains carrying the *AAC (6’)-Ib-cr* gene had a survival advantage.^72^

#### Alterations in efflux pumps

Many efflux pumps have been implicated in fluoroquinolone resistance in *E. coli*, but the AcrAB–TolC system was the first to be reported and has been extensively studied in the past two decades.^79^ The AcrAB–TolC system consists of three structural proteins, AcrA, AcrB, and TolC, which form a tripartite system. Additionally, regulatory proteins such as MarA, Rob, and SoxS are involved in modulating the activity of this efflux pump.^62^ Together, these components form an operon system in which upregulation of the pump leads to innate or acquired resistance, making it the most severe type of efflux-mediated resistance in *E. coli*.^80^ In a 2012 study, upregulation of AcrA and AcrB proteins was identified as the mechanism of resistance in levofloxacin-resistant clinical isolates of *E. coli*. Deletion of AcrA/B proteins significantly increased the intracellular concentration of levofloxacin in *E. coli*, indicating that upregulation of AcrA/B lowers the concentration of levofloxacin and enhances the MIC by 2- to 8-fold, contributing to resistance.^81^

In another study, levofloxacin resistance was induced in 89 *E. coli* isolates, and mRNA levels of AcrA/B were measured. Higher levels of AcrB were found in *E. coli* isolates with moderate and high levofloxacin resistance compared with their susceptible counterparts.^82^ Similar reports have identified upregulation of the structural proteins AcrA and AcrB as contributors to fluoroquinolone resistance, although TolC does not seem to play a significant role.^83,84^ Regulatory proteins also appear to confer fluoroquinolone resistance. In an analysis of 111 non-duplicate isolates of the *E. coli* strain ST131, which exhibited ciprofloxacin and norfloxacin resistance, upregulation of MarA levels was observed compared with control strains, suggesting that this regulator of AcrAB–TolC impacts the activity of the pump and contributes to fluoroquinolone resistance.^85^

#### Alterations in drug permeation

The OmpF porin, located in the outer membrane of *E. coli*, is a 16-stranded β-barrel trimeric protein comprised of a hydrophilic pore that acts a designated entry site for fluoroquinolone influx.^86^ The marR repressor protein and the SoxR regulator protein modulate expression levels of OmpF in *E. coli*, mutations in marR contribute to fluoroquinolone resistance. These mutations reduce the availability of OmpF in the membrane causing a lower intracellular concentration of fluoroquinolone.^87^

Soon after the introduction of norfloxacin in the late 1980s, reports found that *E. coli* K-12 norfloxacin-resistant mutants exhibited a 2-fold decrease in norfloxacin uptake compared with the wild-type parent control.^88,89^ Similar results were observed in 19 *E. coli* isolates with high levels of ciprofloxacin resistance. Researchers utilized SDS-PAGE to assess the expression of porins and found that OmpF was present in 73.7% of isolates. Reduced levels of OmpF were associated with lower intracellular ciprofloxacin concentrations.^69,90^ More recent studies have recognized that OmpF-mediated reduction in ciprofloxacin susceptibility is primarily attributed to mutations in its regulatory genes. Mutations in marR or SoxR were identified in 13 mutants of the *E. coli* strain J53, resulting in a 25% reduction in OmpF expression. Downregulation of OmpF led to decreased ciprofloxacin susceptibility in J53.^91^ Overall, these findings implicate OmpF as a contributor to ciprofloxacin and norfloxacin resistance in *E. coli*. However, alterations in OmpF expression do not affect the MIC levels of fluoroquinolones such as tosufloxacin or sparofloxacin in norfloxacin-resistant *E. coli* isolates. It is possible that these fluoroquinolones traverse the membrane through a different mechanism, thereby avoiding this particular resistance mechanism.^87^ Further investigations would be appropriate to determine whether this is also the case for fourth-generation fluoroquinolones.

### Acinetobacter baumannii

#### Mutations in DNA gyrase and topoisomerase IV

The first reports of *A. baumannii* resistance to fluoroquinolones emerged in the early 1990s. Prior to this, fluoroquinolones exhibited good activity against *A. baumannii* and outperformed other antibiotics such as aminoglycosides or cephalosporins.^92^ In a study investigating 56 cases of *A. baumannii* clinical isolates collected between 2004 and 2006, the QRDR region of GyrA was sequenced, revealing the presence of the S83L mutation in all 56 isolates. Additionally, MIC testing demonstrated that the S83L mutant isolates were resistant to ciprofloxacin, levofloxacin, gemifloxacin, and gatifloxacin.^93^ Similar findings have been reported globally. Ciprofloxacin and levofloxacin-resistant *A. baumannii* isolates have also been found in Egypt. In this study, it was demonstrated that the S83L mutation in the QRDR is a major contributor to fluoroquinolone resistance in *A. baumannii*. However, it was also shown that other, less common mutations can lead to higher levels of fluoroquinolone resistance.^94,95^ In contrast to the high frequency of mutations observed in the canonical dyad between S83 and D87 in *E. coli*, there is limited supporting evidence for the presence of the D87N mutation in *A. baumannii*. For instance, a study examining the mutational profile of 50 ciprofloxacin-resistant clinical isolates of *A. baumannii* using PCR sequencing revealed that 88% of isolates exhibiting full ciprofloxacin resistance harboured the S83L mutation. However, no evidence of the D87N mutation was found, suggesting that the S83L mutation alone may be sufficient to confer high levels of fluoroquinolone resistance in *A. baumannii*, or that other, less common, mutations contribute to higher levels of resistance in this species.^92,96^

Secondary mutations contributing to fluoroquinolone resistance in *A. baumannii* have been identified in the ParC subunit of topoisomerase IV. Specifically, the S80L mutation has been detected in *A. baumannii* isolates, along with the S83L mutation in GyrA of DNA gyrase. It appears that mutations in GyrA serve as primary contributors to fluoroquinolone resistance, while mutations in ParC act as secondary mutations that enhance the level of resistance.^94^ The S80L mutation has also been reported in other studies investigating *A. baumannii* isolates resistant to ciprofloxacin, highlighting its role as a secondary mechanism of resistance.^97^ These findings indicate that ParC mutations contribute to higher levels of fluoroquinolone resistance in this species.^93^

#### Plasmid-mediated quinolone resistance

The isolation of *qnr* genes in *A. baumannii* was first reported in 2008 in non-susceptible isolates to nalidixic acid and norfloxacin.^98^ In an assessment of 100 clinical isolates of *A. baumannii*, 86% were resistant to ciprofloxacin. Subsequent PCR screening for PMQR genes revealed that *qnrA* was present in 66.27% of the isolates, while *qnrS* was present in 70.73% of the isolates.^95^ Although other studies have shown the existence of multiple *qnr* genes in fluoroquinolone-resistant *A. baumannii* isolates, the importance of *qnrA* in conferring fluoroquinolone resistance has been supported by Moosavian et al. In their study, PCR analysis of 105 fully ciprofloxacin-resistant *A. baumannii* isolates showed that *qnrA* was present in 52.6% of the isolates, while *qnrS* was only detected in 3.2% of the isolates and *qnrB* was not detected at all. This suggests that *qnrA* is a more significant factor in conferring ciprofloxacin resistance in this species compared with *qnrB* and *qnrS*.^99^ This idea was further supported by another study that showed only 2.2% of 45 *A. baumannii* isolates resistant to ciprofloxacin expressed *qnrB* or *qnrS*. The low levels of *qnrS* and *qnrB* indicate that these *qnr* genes are unlikely to be the primary mechanism of fluoroquinolone resistance.^100,101^ The consensus from these studies suggests that *qnrA* is predominantly expressed in *A. baumannii*. Furthermore, considering that plasmids carrying *qnr* genes confer low levels of fluoroquinolone resistance, it is likely that they induce other chromosomal mechanisms of resistance to confer full resistance in bacteria.^95^

A high occurrence of *AAC (6’)-Ib-cr* has been reported in clinical isolates of *A. baumannii*, highlighting its significant role in the development of fluoroquinolone resistance in this species. In a study investigating 45 non-duplicate *A. baumannii* isolates with ciprofloxacin resistance, it was found that 46.6% of these resistant isolates also expressed *AAC (6’)-Ib-cr.*^100^ This finding is consistent with another study that revealed the presence of *AAC (6’)-Ib-cr* in *A. baumannii* isolates resistant to levofloxacin and ciprofloxacin. In this study, 74% of the isolates harboured *AAC (6’)-Ib-cr*, which is believed to confer a low level of resistance by acylating fluoroquinolones and impairing their activity.^102^

#### Alterations in efflux pumps

The AdeABC efflux pump was initially reported in a MDR strain of *A. baumannii* (BM4454) by Magnet *et al*. in 2001. Since then, it has been extensively studied and identified as the predominant efflux system contributing to fluoroquinolone resistance in *A. baumannii*.^65,103^ This tripartite system consists of three proteins: AdeA, AdeB, and AdeC. An investigation of 14 clinical isolates of *A. baumannii*, all showing fluoroquinolone resistance, revealed that 71% of the isolates exhibited a 20-fold upregulation of the *AdeB* gene. Researchers confirmed that a single or multiple amino acid substitutions in the regulatory components, AdeRS/AdeN/AdeL, led to the upregulation of AdeABC.^102^ Numerous studies have emphasized the impact of regulatory subunits in modulating the activity of AdeABC and their contribution to the development of fluoroquinolone resistance. Among these studies, more than 85% of *A. baumannii* isolates displayed resistance to ciprofloxacin, levofloxacin, and moxifloxacin.^102,104,105^ The high levels of fluoroquinolone resistance observed in *A. baumannii* are concerning, and understanding the factors that contribute to this resistance can aid researchers in addressing the problem more effectively. In 2018, investigators conducted a phenotypic assay on ciprofloxacin-resistant *A. baumannii* isolates, which revealed that overactive efflux systems were reducing ciprofloxacin susceptibility in the species and influencing the level of resistance. For instance, isolates with a 12-fold increase in AdeB expression exhibited high levels of ciprofloxacin resistance, while isolates with a 4-fold increase displayed intermediate levels of resistance.^105^

#### Alterations in drug permeation

Progress in understanding the contribution of porins to fluoroquinolone resistance in *A. baumannii* has been slow. Among the porins investigated, OmpA has received the most attention in this species. OmpA, also known as HMP-AB, shares homology with OmpF in *P. aeruginosa* and has been found to exhibit low permeability to fluoroquinolones.^66^ OmpA consists of an 8-stranded β-barrel domain that interacts non-covalently with peptidoglycan, which confers its porin activity.^106^

Characterization studies using reconstitution assays have demonstrated that OmpA exhibits comparable levels of slow pore-forming activity to its homologous porin in *P. aeruginosa*. These studies have also confirmed that OmpA is the primary porin in *A. baumannii* and contributes to its intrinsic resistance to fluoroquinolones.^107^ However, further research is needed to fully characterize OmpA and understand its specific role in conferring resistance to different fluoroquinolones.^66^

### Klebsiella pneumoniae

#### Mutations in DNA gyrase and topoisomerase IV

Early reports indicated that mutations at positions 83 and 87 in GyrA of the *K. pneumoniae* strain, ATCC 13883, resulted in decreased susceptibility to ciprofloxacin.^108^ Over the past decade, an increasing number of studies have been published on fluoroquinolone resistance in this species. These studies have demonstrated that *K. pneumoniae* isolates exhibit resistance to levofloxacin, ciprofloxacin, and prulifloxacin.^109^ In another study, 110 non-duplicate *K. pneumoniae* isolates resistant to ciprofloxacin were tested for resistance to other fluoroquinolones. It was found that 88% of the isolates exhibited high resistance not only to ciprofloxacin but also to levofloxacin and ofloxacin.^110^ These reports underscore the fact that isolates resistant to one fluoroquinolone often show cross-resistance to other fluoroquinolones, emphasizing the importance of comprehensive testing for identification and monitoring. Similar to *E. coli* and *A. baumannii*, the S83L mutation has been frequently identified in clinical isolates of *K. pneumoniae* resistant to levofloxacin, ciprofloxacin, nalidixic acid, and gatifloxacin.^110,111^ Furthermore, it appears that a single mutation alone is sufficient to confer resistance, and the accumulation of single amino acid substitutions in GyrA contributes to the development of higher levels of resistance to fluoroquinolones.^108^ This double mutation is not only prevalent in clinical isolates but has also been identified in ciprofloxacin-resistant ecological isolates of *K. pneumoniae* from a wastewater treatment plant in Northern Africa. In these isolates, S83 was mutated to isoleucine, which confers fluoroquinolone resistance similar to the S83L mutation, emphasizing the conserved role of a single amino acid substitution at position 83 in conferring resistance.^112^

Resistance to fluoroquinolones was assessed in 100 clinical isolates of *K. pneumoniae* using the Kirby–Bauer disk diffusion method. The results revealed that 50%, 54%, and 70% of isolates displayed resistance to levofloxacin, ciprofloxacin, and moxifloxacin, respectively. Mutational analysis of ParC identified the S80I mutation as the most prevalent in the resistant isolates, although it conferred a low level of fluoroquinolone resistance when occurring in isolation.^113^ A similar observation was made in a study involving 12 *K. pneumoniae* isolates resistant to ciprofloxacin and prulifloxacin, all of which harboured the S80I mutation, which was absent in the fluoroquinolone-susceptible parent strains.^109^ It should be noted that the small sample size of the latter study raises concerns regarding the generalizability of the findings. However, larger-scale studies conducted more recently have reported similar outcomes, further supporting the role of the S80I mutation in fluoroquinolone resistance.^114,115^

#### Plasmid-mediated quinolone resistance

Unlike other species, the most common *qnr* gene in *K. pneumoniae* is *qnrB*, as supported by several studies.^110,116,117^ This was confirmed in the analysis of eight ciprofloxacin-resistant *K. pneumoniae* isolates, where five isolates expressed *qnrB*, while only three expressed *qnrS*. It is important to note that this study is limited by the low number of isolates, but it has helped confirm the expression of *qnr* genes in this species and has shown a higher occurrence of *qnrB* compared with other *qnr* genes.^118^ Larger studies, such as the one conducted by Geetha *et al.* have provided further evidence and confirmed the reproducibility of the findings from the 2008 study. Using 110 non-duplicate isolates with resistance to ciprofloxacin, levofloxacin, and ofloxacin, the researchers found that 13% of the isolates contained *qnrB*, while only 4.5% harboured *qnrS*. These data emphasize that *qnrB* appears more frequently than other *qnr* genes in *K. pneumoniae*. However, it is important to note that the occurrence of *qnrB* still remains low, indicating that *qnrB* is not the major contributor to full fluoroquinolone resistance, but rather contributes to low levels of resistance in this species.^110,116,118^

In a study conducted in 2015, among 79 *K. pneumoniae* isolates, 38%, 43%, and 45.6% exhibited resistance to levofloxacin, ofloxacin, and nalidixic acid, respectively. Among these resistant isolates, 53.2% expressed the *AAC (6’)-Ib-cr* gene.^119^ This association between fluoroquinolone resistance and *AAC (6’)-Ib-cr* expression has also been observed in *K. pneumoniae* isolates with high levels of ciprofloxacin resistance. In such isolates, 89% were found to harbour the *AAC (6’)-Ib-cr* gene. These findings collectively indicate a high occurrence of *AAC (6’)-Ib-cr* in fluoroquinolone-resistant *K. pneumoniae* isolates.^110,119,120^ Similar results were reported in a 2009 study, where isolates carrying *AAC (6’)-Ib-cr* exhibited a 2-fold increase in the MIC of ciprofloxacin, indicating its role in reducing the activity of fluoroquinolones and decreasing susceptibility.^121^

#### Alterations in efflux pumps

The AcrAB–TolC efflux pump plays a large part in resistance to fluoroquinolones and other antibiotics, such as cephalosporins and erythromycin. It is a tripartite system composed on AcrA, AcrB, and TolC proteins.^122^ AcrAB–TolC has been identified as the predominant efflux pump in a report of 106 non-duplicate fluoroquinolone-resistant *K. pneumoniae* isolates. Its prevalence, compared with other efflux pumps in this species, suggests its involvement in fluoroquinolone resistance.^123^ A study investigating seven ciprofloxacin and nalidixic acid-resistant clinical isolates of *K. pneumoniae* amplified AcrA using PCR and performed immunoblotting with AcrA-specific antibodies. The results showed a correlation between the expression of AcrAB–TolC and fluoroquinolone resistance. All strains exhibited upregulation of AcrA, with up to a 130% increase in expression observed in four isolates compared with their parent strains.^122^

Furthermore, the use of isogenic knockouts lacking the AcrR repressor and AcrB protein in virulent strains of *K. pneumoniae* demonstrated the contribution of AcrB to the development of fluoroquinolone resistance. The knockout strains exhibited significantly higher susceptibility to norfloxacin, ciprofloxacin, and levofloxacin compared with the wild-type controls.^124^ Recent research is building upon these previous findings and investigating the different variants of AcrB that have evolved over time. This emerging understanding suggests that these variants may contribute to enhanced levels of antibiotic resistance in *K. pneumoniae*, leading to more effective efflux pumps and decreased fluoroquinolone susceptibility.^125^

#### Alterations in drug permeation

The major porin channels in *K. pneumoniae*, namely OmpK35 and OmpK36, exhibit homology to the OmpF and OmpC porins in *E. coli*, respectively. Structurally, both OmpK35 and OmpK36 share a trimeric 16-stranded β-barrel architecture.^126,127^ In the investigation of *K. pneumoniae* isolates displaying resistance to ciprofloxacin and nalidixic acid, the role of OmpK35 in fluoroquinolone resistance was examined. It was observed that resistant isolates showed reduced expression of OmpK35, which was suggested to contribute to lower susceptibility to these fluoroquinolones. However, it should be noted that reduced expression or loss of OmpK35 alone is not the sole contributor to resistance, as mutations in GyrA have a more significant impact on reducing susceptibility. Therefore, it is likely that OmpK35 confers fluoroquinolone resistance in conjunction with other mechanisms.^128^

Similar findings have been reported regarding OmpK36 in the ciprofloxacin-resistant MS6671 strain of *K. pneumoniae*. Mutations in the genes encoding OmpK36 were observed, along with a novel gene variant that led to a different amino acid sequence in loop 3 of the OmpK36 eyelet. The eyelet region controls the size of solutes that can bypass the porin, and a different sequence in this region could restrict the entry of ciprofloxacin into the cell, thus conferring resistance.^129^ Furthermore, in the *K. pneumoniae* CSUB10R strain lacking both OmpK35 and OmpK36, the induced expression of OmpK35 resulted in an 8-fold increase in the MIC of ciprofloxacin, compared with the control. These results highlight that the presence of OmpK35 facilitates the transport of fluoroquinolones, enabling their action within the cytosol.^126^

### Pseudomonas aeruginosa

#### Mutations in DNA gyrase and topoisomerase IV

One of the first reports by Yonezawa *et al.* identified single amino acid substitutions in *P. aeruginosa* that contribute to fluoroquinolone resistance. They observed the presence of a D87N and Thr83Ile mutation, which are homologous to the S83L mutation in *E. coli*. The identification of these double mutants provided evidence for the existence of the canonical dyad in *P. aeruginosa* and its role in forming the quinolone binding pocket. Moreover, these mutations demonstrated that similar mechanisms of fluoroquinolone resistance exist across different species.^130^ Several studies have further highlighted the significance of the D87N mutation in conferring fluoroquinolone resistance, particularly in ciprofloxacin- and levofloxacin-resistant *P. aeruginosa* isolates.^50,131^ However, by using Sanger sequencing of the QRDR of GyrA, Zhao *et al.*^132^ demonstrated that the S83L mutation was the most important contributor to resistance in the ciprofloxacin- and levofloxacin-resistant *P. aeruginosa* strain ATCC 27853.

Analysis of the QRDR of ParC, using Sanger sequencing and pyrosequencing, revealed that the most prevalent mutation in ciprofloxacin-resistant *P. aeruginosa* isolates was the substitution of serine 80 with leucine (S80L). Additionally, it was observed that this position can also be mutated to a tryptophan residue (S80W).^133^ These findings are supported by another study, which identified S80L/W mutations in ParC in seven ciprofloxacin-resistant isolates of *P. aeruginosa*. Collectively, these data suggest that the mutation of serine to either leucine or tryptophan is associated with ciprofloxacin resistance in *P. aeruginosa*.^133,134^

#### Plasmid-mediated quinolone resistance

The prevalence of *qnr* genes in *P. aeruginosa* isolates varies depending on the geographical location. For example, in Saudi Arabia, the most common *qnr* gene in fluoroquinolone-resistant *P. aeruginosa* isolates is *qnrS* with a study identifying 79.5% of 92 tested isolates positive for *qnrS*, while *qnrA* and *qnrB* were not detected in any of the isolates showing resistance to various fluoroquinolones.^117^ However, in Iran, *qnrB* has been reported as the most prevalent gene in *P. aeruginosa* isolates exhibiting resistance to gatifloxacin, ofloxacin, levofloxacin, ciprofloxacin, and norfloxacin.^135^ These differences in the prevalence of *qnr* genes based on geographical location highlight the variation in resistance profiles of *P. aeruginosa*. Surveillance programmes play a crucial role in assessing these differences in fluoroquinolone resistance, understanding how they arise, and determining the most appropriate antibiotic treatment based on the resistance profiles specific to each country.^117,135^

*AAC(6’)-Ib-cr* has not been reported as frequently in *P. aeruginosa* compared with other species such as *E. coli*.^136^ However, more recent studies have provided supporting evidence for the presence of *AAC(6’)-Ib-cr* in conferring fluoroquinolone resistance in this species. For example, a study using PCR and sequencing methods confirmed that all 100 fluoroquinolone-resistant *P. aeruginosa* isolates tested contained the *AAC(6’)-Ib-cr* gene. Interestingly, no *qnr* genes were identified, suggesting that *AAC(6’)-Ib-cr* is the predominant contributor to PMQR-mediated fluoroquinolone resistance in *P. aeruginosa*.^137^ Another study also noted a high occurrence of *AAC(6’)-Ib-cr* in ciprofloxacin-resistant *P. aeruginosa* isolates.^100^ However, contrasting results were observed in the study by Saki et al. Their investigation of 185 clinical isolates of *P. aeruginosa* resistant to ciprofloxacin (59.4%) or ofloxacin (45.9%) did not confirm the presence of *AAC(6’)-Ib-cr.* It is noteworthy that the isolates in the Saki et al. study were from southwest Iran, while the study by Molapour et al. involved isolates from northern Iran. These differing outcomes emphasize that fluoroquinolone resistance can be mediated through different mechanisms, even within the same species, and that the prevalence of resistant genes can vary among isolates from different geographical locations.^135,137^

#### Alterations in efflux pumps

MexAB-OprM is the only known efflux pump in *P. aeruginosa* that is constitutively expressed at a high enough level to cause intrinsic fluoroquinolone resistance. This tripartite system is composed of MexA, MexB, and OprM proteins.^138^

Researchers have demonstrated that 11 strains of norfloxacin-resistant *P. aeruginosa* exhibited higher levels of MexAB-OprM mRNA compared with the control strain, PA01.^139^ Similar findings were observed in *P. aeruginosa* isolates induced with ciprofloxacin resistance (20 mg/kg/day), where OprM expression was increased by 2- to 3-fold after four days compared with their fluoroquinolone-susceptible counterparts.^140^ Pharmacological inhibition of MexAB-OprM in the levofloxacin-resistant strain PAM1537 (MIC 8 mg/L) resulted in a 32-fold reduction in resistance (MIC 0.25 mg/L). According to the EUCAST breakpoint guidelines, MIC values >2 mg/L indicate resistance. This finding contributes to the understanding that MexAB-OprM plays a role in fluoroquinolone resistance, and inhibiting its activity can decrease the emergence of new *P. aeruginosa* strains with resistance.^141^ Additionally, other mechanisms of resistance, such as mutations in DNA gyrase, can further enhance the level of fluoroquinolone resistance in *P. aeruginosa*. By constructing cells with double or single mutations in GyrA and/or MexR, researchers demonstrated that double mutant *P. aeruginosa* bacterial cells exhibit 1024 times higher fluoroquinolone resistance compared with single mutants.^142^

#### Alterations in drug permeation

OprF serves as the primary porin in *P. aeruginosa* and plays a significant role in conferring fluoroquinolone resistance in this species. This porin consists of an N-terminal β-barrel domain and a C-terminal periplasmic α-helical domain.^143^ Compared with OmpF in *E. coli*, OprF exhibits approximately half the permeability to fluoroquinolones. This inherent lower permeability contributes to the intrinsic resistance to fluoroquinolones mediated by OprF in *P. aeruginosa*.^49^

In the mid-1990s, early studies began investigating OprF and its role in fluoroquinolone resistance. It was demonstrated that the *P. aeruginosa* strain G49, when induced to become resistant to enoxacin, ciprofloxacin, and nalidixic acid, could completely lose OprF.^144^ Since then, there has been significant research focused on understanding how the low permeability of OprF contributes to fluoroquinolone resistance, particularly considering the serious infections caused by *P. aeruginosa*. Advancements in computational and structural biology over the past decade have allowed researchers to use X-ray crystallography to confirm that OprF predominantly exists in a closed state. This has led to the conclusion that the slow conduction of OprF is attributed to its poor permeability, as channels are closed approximately 95% of the time. These findings have provided insights into how the low permeability of OprF confers high intrinsic resistance to fluoroquinolones.^106^

### Membrane remodelling

Although the role of membrane remodelling in conferring fluoroquinolone resistance has not been extensively studied, it is important to mention its potential contribution. Membrane remodelling in Gram-negative bacteria refers to the modifications made to the membrane proteome, including the degradation of existing proteins and the insertion of new proteins. These changes can lead to the loss of porins in the membrane, which in turn contributes to antibiotic resistance.^145^ The composition of the membrane proteome can be influenced by various cellular environments during remodelling, which can affect the abundance of specific porins in the membrane of *E. coli*. Inducing different conditions can result in different proportions of OmpF or OmpC in the membrane. For example, when OmpF is completely lost, increased resistance to norfloxacin has been observed.^146^ Similar findings have been noted in *K. pneumoniae*, where forced membrane remodelling led to different levels of OmpK35 and OmpK36, ultimately resulting in antibiotic-resistant phenotypes.^147^

## Overcoming fluoroquinolone resistance

There has been a recent surge in the scientific community to investigate different approaches that can be taken to overcome the mechanisms of fluoroquinolone resistance associated with Gram-negative bacteria. Multiple strategies have been tested to develop novel therapies that can bypass the currently known mechanisms of resistance. These strategies can be classified into two main categories:

- Development of novel agents: Scientists are exploring the development of new compounds that can maintain their activity against DNA gyrase/topoisomerase IV, the target enzymes of fluoroquinolones, by forming different interactions than the conventional water-metal ion bridge. By designing compounds that can effectively bind to these enzymes and inhibit their activity, researchers aim to overcome resistance mechanisms and restore the effectiveness of fluoroquinolones.^148–150^
- Potentiation of existing fluoroquinolones: Another approach involves the development of compounds that can enhance the actions of existing fluoroquinolones. These compounds, known as adjuvants or potentiators, work in combination with fluoroquinolones to increase their efficacy. By targeting specific resistance mechanisms or pathways, these compounds can overcome or bypass the resistance mechanisms employed by Gram-negative bacteria and restore the potency of fluoroquinolones. These strategies represent promising avenues for the development of new therapies against fluoroquinolone-resistant Gram-negative bacteria. Continued research and innovation in this field are essential to address the challenge of antibiotic resistance and ensure effective treatment options for bacterial infections.^151–154^

### Design of antimicrobial agents that retain activity against target enzymes

Mutations in DNA gyrase/topoisomerase IV have been established as the primary mechanism of resistance in Gram-negative bacteria, reducing the efficacy of fluoroquinolones and conferring resistance. Consequently, the development of novel fluoroquinolones that target these enzymes through different interactions has been proposed to retain activity and enhance efficacy.^148^ Initially, this approach was considered impractical, as different mutations in the target enzymes were assumed to confer resistance through different mechanisms. However, this assumption was disproved, and it was confirmed that the most common mutations disrupt the water-metal ion bridge, which is essential for resistance.^32^

Researchers have investigated the role of common mutations in GyrA and ParC, as mentioned earlier, in relation to the water-metal ion bridge. These mutations increase the minimum concentration of Mg^2+^ ions required for DNA cleavage and completely eliminate interactions between the enzyme and fluoroquinolones. Characterization studies have also confirmed the presence of the water-metal bridge and its significance as the primary interaction between the target enzymes and fluoroquinolones.^37,38^ The correction of this misconception has led to a unanimous consensus that the design of novel fluoroquinolones targeting DNA gyrase/topoisomerase IV without relying on the water-metal ion bridge can overcome target-mediated resistance.^32^

#### Quinazolinediones

**Figure 5.**
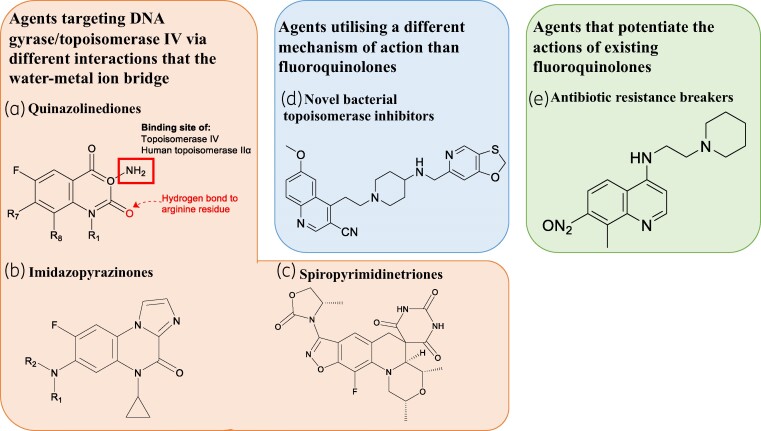
(a) Quinazolinediones core shows lacks the keto acid moiety on quinazolinediones that usually forms the water-metal ion bridge in fluroquinolones.^20^ (b) Skeletal structure of the novel class of tricyclic imidazopyrazinones which lack the carboxylic acid moiety at the C3 present in fluoroquinolones.^155^ (c) Skeletal structure of the lead spiropyrimidinetrione of this class, AZD0914 (zoliflodacin) contains a benzisoxazole scaffold and a spirocyclic pyrimidinetrione core.^150^ (d) NBTIs contain a head group (left-hand side) which binds DNA and a tail region (right-hand side) that interacts with the target enzymes. These two regions are enjoined by a linker section.^154^ (e) Compound 814 is an alkylaminoquinolone derivative developed as an EPI to prevent the efflux of fluoroquinolones.^153^ Figure created with ChemDraw.

Quinazolinediones have emerged as potential candidates for the design of novel antimicrobial agents that maintain activity against DNA gyrase/topoisomerase IV. Unlike fluoroquinolones, quinazolinediones do not possess the keto acid moiety that is involved in the water-metal ion bridge formation. Instead, they feature a carbonyl group on C2, which forms a hydrogen bond with a conserved arginine residue in the target enzyme (Figure 5a).^148^ This alternative interaction allows quinazolinediones to bypass the mechanism of resistance associated with the disruption of the water-metal ion bridge, making them promising candidates for combating resistant Gram-negative bacteria.

Quinazolinediones have demonstrated comparable or even higher levels of activity against DNA gyrase/topoisomerase IV, particularly in the presence of mutations in serine or aspartic acid residues, when compared with clinically available fluoroquinolones.^20^ This enhanced activity can be attributed to the hydrogen bond interaction between the carbonyl group at C2 of quinazolinediones and the glutamine residue in the binding pocket, as supported by structural evidence.^32,156^ Studies investigating the effect of quinazolinediones on the ciprofloxacin and moxifloxacin-resistant *E. coli* K-12 S83 mutant strain have demonstrated similar or even higher susceptibility levels compared with the wild-type control. These findings confirm that this lead structure can maintain its activity against fluoroquinolone target enzymes and hold promise in terms of efficacy against bacterial mutant cells.^148^

Quinazolinediones have demonstrated potential in overcoming fluoroquinolone resistance. However, their main limitation lies in the relatively weak interaction formed in the binding pocket, unless there are additional groups to strengthen the interaction with the cleavage complex. Quinazolinediones with a C7 3′-(aminomethyl)pyrrolidinyl substituent in their pharmacophore have shown greater activity due to stronger interactions in the binding pocket. However, it is important to note that this substituent also interacts with human topoisomerase IIα, which raises concerns about potential cross-reactivity with human enzymes.^32^ Therefore, the identification and modification of substituents that interact specifically with bacterial enzymes while avoiding interactions with human topoisomerase IIα is crucial. Achieving this balance can be challenging, as it is necessary to retain activity against fluoroquinolone-resistant bacterial enzymes. Nonetheless, recent progress has been made in the synthesis of quinazoline-2,4-diamine analogues that exhibit efficacy against *A. baumannii*, indicating promising developments in this area.^157^

#### Imidazopyrazinones

Another class of compounds developed to target bacterial DNA gyrase/topoisomerase without relying on the water-metal ion bridge are imidazopyrazinones. Unlike fluoroquinolones, imidazopyrazinones do not possess a carboxylic acid moiety. However, they adopt a similar conformation to fluoroquinolones within the binding pocket of DNA gyrase/topoisomerase IV, thereby stabilizing the DNA-enzyme complex (Figure 5b).^149^

Imidazopyrazinones initially demonstrated potential in their activity against Gram-negative bacteria. However, the discovery of partial cross-resistance with fluoroquinolones led to the discontinuation of lead optimization efforts. Suggestions were made to modify the core structure of fluoroquinolones to more closely resemble imidazopyrazinones, such as restructuring the core rings into the tricyclic core of imidazopyrazinones.^155^ Unfortunately, little progress has been made with imidazopyrazinones, and this class serves as an example of the challenges encountered in developing new strategies to overcome fluoroquinolone resistance.^158^

#### Spiropyrimidinetriones

Spiropyrimidinetriones represent a novel class of antibiotics that function similarly to fluoroquinolones by inducing double-stranded breaks in DNA through enzyme mediation. One notable compound in this class is AZD0914, also known as zoliflodacin, which serves as the original lead structure. AZD0914 features a benzisoxazole scaffold and a spirocyclic pyrimidinetrione core (Figure 5c).^150^ AZD0914, similar to fluoroquinolones like ciprofloxacin, acts by inhibiting DNA gyrase-mediated supercoiling, decatenation by topoisomerase IV, and stabilizing the cleavage complex formed with the target enzyme. It has demonstrated potent activity against Gram-negative bacteria, including fluoroquinolone-resistant mutants of *P. aeruginosa*.^150^  *In vitro* susceptibility testing of AZD0914 against 53 ciprofloxacin-resistant *E. coli strains* revealed MIC values between 0.125 and 16 mg/L, and AZD0914 demonstrated no difference in antibacterial activity between ciprofloxacin-resistant or susceptible isolates.^159^

The distinguishing feature of AZD0914 is its ability to form a different interaction, which does not rely on chelating interactions with magnesium ions, unlike fluoroquinolones.^160^ Instead, it is proposed to interact with residues in GyrB, rather than forming the water-metal ion bridge in GyrA, to stabilize the DNA cleavage complex. This unique interaction mechanism explains AZD0914's ability to maintain its activity against fluoroquinolone-resistant species without showing cross-resistance.^160,161^ Studies have reported significantly lower modal MIC values with AZD0914 (MIC 0.125 mg/L) compared with ciprofloxacin (MIC >32 mg/L). The enhanced susceptibility of AZD0914 supports its potential as a novel approach to target fluoroquinolone-resistant mutants. Currently, AZD0914 is undergoing Phase II/III clinical trials for the treatment of rectal infections.^150,162^

### Novel bacterial topoisomerase inhibitors

Novel bacterial topoisomerase inhibitors (NBTIs) represent a novel class of type II topoisomerase inhibitors that offer a different mechanism of action compared with existing fluoroquinolones. This unique mechanism provides a promising strategy to overcome target-mediated resistance commonly observed with fluoroquinolones.^154^ One advantage of NBTIs is their ability to bypass cross-resistance mechanisms associated with fluoroquinolones, making them attractive candidates for developing potent agents against multiple MDR bacterial species.^151^

Structurally, NBTIs consist of a head group that binds to DNA, a tail region that interacts with the target enzymes, and a linker section that connects these two regions (Figure 5d).^163^ This unique architecture allows NBTIs to interact with the topoisomerase enzymes in a distinct manner, providing an opportunity to overcome resistance mechanisms associated with traditional fluoroquinolones. Further research and development efforts are underway to optimize and evaluate the efficacy of NBTIs as promising therapeutic agents against MDR bacteria.^151,163^

Unlike fluoroquinolones, which typically require two molecules to bind to the active site, molecular docking simulations have shown that a single NBTI molecule can bind effectively between the bonds undergoing scission in the cleavage complex. Additionally, it has been suggested that a secondary hydrogen bond can form between an acidic residue and the NBTI, potentially enhancing its activity.^151,164^ NBTIs exert their inhibitory effect on bacterial type II topoisomerases by stabilizing the enzyme-DNA complex with only one-strand break, in contrast to fluoroquinolones which stabilize double-strand DNA breaks. This unique mechanism allows NBTIs to maintain high levels of activity against bacteria, including those exhibiting target-mediated fluoroquinolone resistance. The efficacy of a novel NBTI developed by Redx Pharma has been demonstrated in fluoroquinolone-resistant isolates of *A. baumannii* and *E. coli*, highlighting the potential of NBTIs as effective therapeutic agents. Further research is underway to explore and optimize the activity and selectivity of NBTIs for clinical applications.^163,164^

Indeed, further research is crucial to fully understand the activity and efficacy of NBTIs, especially in the context of purified mutants of DNA gyrase and topoisomerase IV with fluoroquinolone resistance. These studies will help elucidate the potential of NBTIs in overcoming resistance mechanisms.^165,166^ Despite the ongoing research, some NBTIs have already shown promise and have progressed to clinical trials. One notable example is gepotidacin, a novel NBTI that has reached Phase III clinical trials for the treatment of skin-related bacterial infections.^164^ Additionally, gepotidacin is being investigated for its effectiveness against other bacterial infections caused by Gram-negative bacteria. Gepotidacin has shown antimicrobial activity against *Stenotrophomonas maltophilia*, a pathogen with MDR to fluroquinolones, carbapenems, aminoglycosides, and others*. In vitro* susceptibly testing on 99 *S. maltophilia* isolates against gepotidacin revealed MICs between 0.25 and 16 mg/L (MIC_50_: 2 mg/L; MIC_90_: 8 mg/L).^166^ The development and advancement of NBTIs like gepotidacin highlight the potential of this class of antibiotics as alternative treatment options. As clinical trials progress, more information will be gathered regarding the safety, efficacy, and potential applications of NBTIs in the treatment of various bacterial infections.^165^

### Other novel approaches

Antibiotic resistance breakers (ARBs) are compounds specifically designed to overcome resistance mechanisms associated with certain antibiotics. In the case of fluoroquinolones, ARBs are molecules that enhance the effectiveness of these antibiotics. They can be directly attached to the fluoroquinolone molecule or administered in combination to counteract specific resistance mechanisms.^152,153^

Efflux pump inhibitors (EPIs) are a type of ARB that has been developed to potentiate the activity of fluoroquinolones in Gram-negative bacteria that exhibit efflux-mediated resistance. As mentioned earlier, efflux pumps can reduce the intracellular concentration of fluoroquinolones by actively pumping them out of bacterial cells, leading to decreased efficacy. To address this issue, EPIs have been designed to inhibit the efflux of fluoroquinolones. One example is compound 814, an alkylaminoquinolone derivative, which has been shown to prevent the efflux of norfloxacin and enhance its efficacy against the EA3 strain of Klebsiella aerogenes, a Gram-negative bacterium, by 8-fold (from 128 to 16 mg/L) (Figure 5e).^153,167^

These ARBs, including EPIs, offer a potential strategy to overcome fluoroquinolone resistance by enhancing the activity of these antibiotics and counteracting specific resistance mechanisms. Continued research and development in this field may lead to the discovery of more effective ARBs and EPIs, offering new solutions in the battle against antibiotic resistance.^153,167^

## Future perspectives

The evaluation of fluoroquinolone resistance in Gram-negative bacteria has provided valuable insights into the prevailing mechanisms and trends observed over the past two decades. Mutations in GyrA and ParC have been consistently identified as the predominant forms of fluoroquinolone resistance, even in newer generations of these antibiotics.^68,93^ Similar trends have been observed with other resistance mechanisms, including efflux pumps, porin alterations, and PMQR.^75,117^ However, there have also been reports of novel mutations, such as the L157Y mutation, which raise concerns about the potential development of resistance to newer fluoroquinolone generations.^113^

Despite the wealth of literature on fluoroquinolone resistance, there is a notable information stagnation in terms of understanding the molecular contributors to resistance. The majority of studies recapitulate previous findings related to susceptibility testing, mutational landscapes, and the prevalence of resistance mechanisms. However, there is a lack of understanding regarding the underlying molecular mechanisms that drive fluoroquinolone resistance. Future research should focus on elucidating the impact of different levels of DNA supercoiling on fluoroquinolone susceptibility across species. This knowledge is crucial for the development of newer fluoroquinolone generations that can be targeted to specific levels of supercoiling.^168,169^

Furthermore, there is a clear need for the incorporation of *in silico* technology in investigating fluoroquinolone resistance in Gram-negative bacteria. While older studies relied on PCR and Sanger sequencing, newer technologies such as the CRISPR/Cas9 system offer powerful tools for investigating resistance mechanisms. By utilizing *in silico* approaches, including molecular modelling and simulation, researchers can gain valuable insights into the interactions between fluoroquinolones and their target enzymes. This will not only improve our understanding of resistance mechanisms but also aid in the design of novel fluoroquinolones with enhanced efficacy.^9,170^ As new-generation fluoroquinolones are produced to overcome specific resistance mechanisms in Gram-negative bacteria, precision medicine could be crucial in selecting the most suitable fluoroquinolone based on the resistance mechanism identified in the isolated bacteria. For example, researchers have employed resistance risk factor prediction tools alongside antibiograms to select the most appropriate antibiotic. This approach in precision medicine is expected to offer the most effective and targeted treatment while minimizing the possibility of developing further resistance.^171^

The findings of this review reinforce the notion that current fluoroquinolones will eventually face complete resistance. This realization has driven researchers to explore alternative strategies for combating fluoroquinolone resistance. Cryo-electron microscopy (cryo-EM) is an emerging technology that can provide atomic-level resolution of efflux pump systems. Unlike X-ray crystallography, cryo-EM captures the dynamic nature of proteins and can visualize multiple conformational states. Its application in studying efflux pump systems holds great promise for the design of more potent EPIs based on higher resolution structures.^172^

Using antimicrobial susceptibility testing (AST) to generate a cumulative antibiogram can serve as an AMR surveillance measure offering insight into the rise of AMR and aiding in guiding appropriate clinical decision-making.^173^ Using AST to selectively report susceptibility is a recommended antimicrobial stewardship programme, which helps curb unnecessary and inappropriate antibiotic use.^174,175^

Whole-genome sequencing (WGS) is increasingly being used to detect antibiotic resistance, WGS is able to detect all the genes involved in AMR and shows no discordance in resistance profiles obtained using phenotypic susceptibility testing.^175^ WGS is advancing the field by providing useful insight into the emergence and spread of AMR, for instance WGS has shown the transmittance of *P. aeruginosa* Liverpool strain between clinicals in the UK and North America.^176^ However, slow turnover times, increased cost, and existence of unknown mechanisms of resistance hinder the use of WGS in routine clinical practice.^175^ The current understanding of AMR in Gram-negative bacteria highlights the potential of AST-driven antibiograms for AMR surveillance.

To effectively reduce the spread of fluoroquinolone resistance, it is imperative to further investigate and address unanswered questions surrounding resistance mechanisms. In particular, future research should focus on exploring new technologies and strategies to develop more robust antibiotics. The integration of *in silico* approaches, such as molecular modelling and simulation, can significantly enhance our understanding of AMR and facilitate the design of novel fluoroquinolones with improved efficacy and reduced resistance potential. Additionally, the implementation of effective antibiotic stewardship programmes is essential to promote responsible antibiotic use and minimize the spread of fluoroquinolone resistance.^168–176^

### Conclusion

Fluoroquinolones have been invaluable in treating bacterial infections, but their extensive use has led to increasing levels of resistance in Gram-negative bacteria. The reviewed studies consistently demonstrate that mutations in GyrA and ParC remain the most prevalent forms of fluoroquinolone resistance across different Gram-negative species, geographical locations, and fluoroquinolones used. These mechanisms, along with alterations in porins/efflux pumps and PMQR, are the primary drivers of fluoroquinolone resistance. However, the limited focus on alternative resistance mechanisms in routine studies may have hindered the identification of other mechanisms. Efforts are underway to develop novel strategies to overcome fluoroquinolone resistance in Gram-negative bacteria. ARBs show promise, but regulatory and financial challenges need to be addressed before their clinical use. Two notable treatments, gepotidacin (spiropyrimidinetrione) and zoliflodacin (NBTI), have shown promising results in preliminary studies and are currently in clinical trials, offering hope for the future treatment of fluoroquinolone-resistant Gram-negative bacteria. In conclusion, the increasing resistance of Gram-negative bacteria to fluoroquinolones necessitates the exploration of novel strategies to combat this problem. Understanding the prevailing mechanisms of resistance and identifying alternative targets is crucial. Although challenges remain, the development of ARBs and the promising results from gepotidacin and zoliflodacin in clinical trials provide optimism for the future. Continued research, along with effective antibiotic stewardship programmes, is essential to reduce the spread of fluoroquinolone resistance and preserve the efficacy of this important class of antibiotics.
